# Polyphenols in health and food processing: antibacterial, anti-inflammatory, and antioxidant insights

**DOI:** 10.3389/fnut.2024.1456730

**Published:** 2024-08-19

**Authors:** Shengqian Sun, Zhengyang Liu, Mingxia Lin, Na Gao, Xiaojie Wang

**Affiliations:** ^1^Yantai Key Laboratory of Special Medical Food, School of Food and Bioengineering, Yantai Institute of Technology, Yantai, Shandong, China; ^2^College of Pharmacy, Binzhou Medical College, Yantai, Shandong, China; ^3^Department of Medical Records Management, Yantai Affiliated Hospital of Binzhou Medical University, Yantai, Shandong, China

**Keywords:** bioactive constituents, biological activity, extraction methods, sustainable processing, mechanisms

## Abstract

Polyphenols, as subordinate metabolites of plants, have demonstrated significant antibacterial, anti-inflammatory, and antioxidant action in scientific learn. These compounds exert their effects through various mechanisms, containing interference with microbial cell structures, rule of host immune responses, and neutralization of free radicals. This multifaceted activity positions polyphenols as promising candidates for maintaining human health and treating related diseases. Notably, in the context of escalating antibiotic resistance, the antibacterial properties of polyphenols offer innovative avenues for the development of new therapeutic agents. Additionally, their anti-inflammatory and antioxidant effects hold substantial potential for treating inflammatory diseases and mitigating the aging process. This review aims to summarize the latest findings on the biological activities of polyphenols, highlighting their mechanisms of action and potential applications in health and disease management. Furthermore, optimizing polyphenol extraction methods aligns with the goals of sustainable and green processing, reducing environmental impact while enhancing food safety and extending shelf life. Employing advanced analytical techniques, such as spectroscopy and chromatography, can ensure the accurate evaluation of polyphenol content and efficacy. These efforts collectively contribute to the ongoing improvement of food processing practices and product quality, promoting a healthier and more sustainable future in the food industry.

## 1 Introduction

Polyphenols, which are bioactive compounds synthesized by plants, can be found in many plant structures, from fruits and flowers to seeds, leaves, and woody sections. Polyphenols are highly diverse in their structure and function, with over 8,000 identified phenolic structures ranging from simple phenolic acids to complex, highly polymerized substances such as tannins (1). Polyphenols are characterized by their distinctive structural feature of one or more aromatic rings containing hydroxyl groups and their derivatives. Polyphenols can be classified into several main classes based on the strength of their phenolic ring structure, including phenolic acids, flavonoids, stilbenes, phenolic alcohols, and lignans (2).

The health benefits of polyphenols have garnered significant attention, particularly their antibacterial, anti-inflammatory, and antioxidant properties. These compounds are increasingly linked to clean label products, which appeal to consumers seeking healthier food options (3). The growing consumer preference for natural food additives over synthetic ones, driven by concerns about the potential carcinogenicity of synthetic compounds, has encouraged the food industry to explore natural alternatives (4). Consequently, research on polyphenols is on the rise due to the abundance of plant species in nature, which results in a vast quantity and widespread distribution of these compounds (2, 5). As a result, it is impractical to exhaustively cover every type of plant polyphenol. Despite significant progress in understanding polyphenols, challenges persist in optimizing their extraction and elucidating their pharmacological activities, owing to their immense diversity.

The synthesis, accumulation, and stability of polyphenols in plants are governed by a complex interplay of internal and external factors (Table 1A), these factors significantly impact the polyphenol content, highlighting the need for optimized cultivation, harvesting, and processing methods. Despite advancements, challenges in standardizing polyphenol extraction and maintaining stability persist due to their diverse nature. Addressing these challenges is crucial for improving polyphenols’ health benefits, particularly in replacing synthetic antioxidants in food products, necessitating further research into genetic mechanisms and innovative agricultural technologies. A holistic approach that integrates genetic, environmental, and agricultural factors is essential for maximizing the benefits of polyphenols. This will not only improve their application in the food industry but also enhance their contribution to human health. The objective of this review is to enhance the understanding of polyphenols and illustrate advancements in related disciplines. This article aims to provide an overview of the antibacterial, anti-inflammatory, and antioxidant properties of polyphenols, as well as the factors influencing their content and stability. By consolidating recent research findings, this review seeks to offer valuable insights for future studies and practical applications in health science and food processing technology.

**Table 1:** Comprehensive insights into polyphenol content and stability in plants, extraction optimization, and anti-inflammatory mechanisms: (A) factors influencing polyphenol content and stability in plants; (B) optimization methods for polyphenol extraction: advantages, mechanisms, applications, and challenges; (C) mechanisms of anti-inflammatory action of polyphenols: pathways, factors, and effects.

A: Influence factor	Subfactors	Detail	Influence the result	Reference
Internal factor	Floristics	The quantity and variety of polyphenols present in different plant species is markedly disparate.	Differences in the type and content of polyphenols	(6, 7)
	Parts of plants	The levels of polyphenols present in different parts of the same plant exhibited considerable variation.	The content of polyphenols in the fruit pit was high	(8)
	Grade of maturity	The degree of maturity of the plant has a significant impact on the synthesis and accumulation of polyphenols.	The content of polyphenols increased with the increase of maturity	(9)
	Heritable variation	Genotype differences lead to differences in the efficiency of the polyphenol synthesis pathway	The polyphenol content was affected	(10)
External factor	Environmental	Climatic conditions had a significant effect on polyphenol biosynthesis	Polyphenol synthesis was affected	(10, 11)
	Edaphic condition	Soil factors affect the synthesis and accumulation of polyphenols in plants	Polyphenol synthesis and accumulation were affected	(12)
	Agricultural practice	Agricultural practices such as fertilization and irrigation affect polyphenol content	Changes in polyphenol content	(13, 14)
	Harvest and storage conditions	Harvest and storage conditions affect the stability and content of polyphenols	Free phenolics were reduced	(15)
	Processing method	The choice of processing method can influence the extraction and stability of polyphenols.	In the case of carrots, frying is easier to protect polyphenols	(16–18)
	Post-harvest treatment	The processing, transportation, and storage conditions can lead to the degradation or transformation of polyphenols.	Polyphenol degradation or transformation	(19, 20)

B: Method	Advantage	Explanation	Mechanism	Applications	Challenges	Reference
Solvent selection	Improves extraction efficiency by selecting the most suitable solvent based on polyphenol properties.	Water, ethanol, methanol, and acetone are commonly used. Selection depends on solubility and stability of target polyphenols.	Different solvents dissolve polyphenols to varying degrees.	Used in processes where target polyphenols vary significantly in properties.	Potential toxicity or environmental impact of organic solvents.	(21)
Solvent strength	The extraction rate can be increased and the solvent loss reduced by adjusting the extraction concentration.	Typically, two or more solvent mixtures or single solvents of different concentrations are used.	The interaction of appropriate solvent concentration can change the solubility and stability of polyphenol molecules and improve the extraction efficiency.	It is mainly used to solve the problem of solubility and stability of polyphenols in different concentrations of extracts.	Determining the optimal ratio is difficult.	(22)
Time	The optimal extraction time was determined to increase extraction efficiency and polyphenol stability.	The time of contact between polyphenols and the extraction solution affected the extraction efficiency and solvent utilization.	The extraction process takes a certain amount of time, too short extraction is insufficient, and too long leads to solvent waste.	Optimizing extraction time is mainly used to find ways to reduce cost losses while increasing extraction volume.	To determine the optimal extraction time requires a lot of experiments and high cost.	(21)
Temperature	Temperature is controlled to balance efficiency and stability in the extraction process.	The extraction efficiency and activity of polyphenols are mainly affected by the degree Celsius.	Select the appropriate temperature of the extraction solution to reduce the degradation of heat-sensitive polyphenols and increase energy consumption.	It is used to solve the process that affects the extraction efficiency due to temperature sensitivity.	May increase energy consumption and may accelerate solvent volatilization.	(23)
Solid-to-liquid ratio	Improve extraction efficiency, increase solvent utilization rate and reduce solvent loss.	It mainly refers to the mass ratio or volume ratio, and the appropriate ratio can reduce solvent loss and improve extraction efficiency.	The solid–liquid ratio directly affects the contact area and contact time between polyphenol and solvent, thus affecting the extraction efficiency.	It is used in the process of controlling the extraction rate of the target extract and improving the extraction efficiency.	Improper ratio will lead to difficult solvent recovery and ungraded extraction.	(22)
pH	Adjusting the extraction environment to a suitable pH value can improve the chemical stability of polyphenols and maintain their activity.	At certain pH values, polyphenols may degrade, oxidize, or interact with solvents, resulting in reduced selectivity.	Adjust the appropriate pH to increase the extraction efficiency of target polyphenols, increase the stability of polyphenols and the purity of products.	It is used to solve the solubility and activity problems of target polyphenols.	It may lead to the degradation or polymerization of polyphenols, which will affect the subsequent treatment.	(24)
Assisted extraction technique	On the basis of the original extraction, a new auxiliary extraction technology can be added to obtain more accurate control, higher yield and higher efficiency.	Including ultrasonic, microwave, enzyme and other auxiliary extraction technology, we can choose the appropriate auxiliary extraction technology according to the nature of the target polyphenols.	Some auxiliary extraction techniques can promote the release of polyphenols, reduce the loss of heat-sensitive polyphenols, and accelerate the extraction process and extraction efficiency.	It is generally used to solve the problem of inadequate extraction and low efficiency.	Generally, more complex equipment and steps are required, increasing the difficulty and cost of operation.	(25)

C: Mechanisms of anti-inflammatory action	Detail	Related factors/pathways	Anti-inflammatory effects	Reference
Inhibit the production of inflammatory mediators	Inhibit the production of inflammatory mediators by inflammatory cells	PGE2, TNF-α, IL-1β, IL-6	Reduced production of inflammatory mediators	(26)
Regulatory signaling pathways	Affect intracellular signaling and inhibits NF-κB activation	NF-κB, IKK, MAPK	Reduce inflammation	(27)
Anti-oxidation	Neutralize free radicals and reactive oxygen species and reduce oxidative stress	ROS	Protect cells from damage	(28)
Enzymes activity	It regulates the activity of inflammation-related enzymes	COX, LOX, MAPK, IKK	Inhibit the synthesis of inflammatory mediators	(29)
Modulating the immune response	It affects immune cell populations and cytokine production	Regulatory T cells, Th1 cells	Enhanced anti-inflammatory cell function and inhibited pro-inflammatory cell activity	(30)
Gene expression & transcription factor activation	It inhibited the activation of nuclear transcription factors and reduced the expression of cell adhesion molecules	STAT3, ICAM-1, VCAM-1	Inhibits inflammatory gene expression and cell adhesion	(31, 32)

## 2 Extraction process and determination method

### 2.1 The extraction technology of polyphenols

The extraction technology of polyphenols can be broadly categorized into traditional and modern techniques (Table 1B). Traditional methods, such as impregnation, percolation, and Soxhlet extraction, are commonly employed in phytochemical analysis due to their straightforward operational characteristics. However, these methods often involve lengthy processing times, high costs, and the use of environmentally unfriendly solvents, which are significant drawbacks (33).

In contrast, modern extraction technologies, including ultrasound-assisted extraction, microwave-assisted extraction, and supercritical fluid extraction, provide notable enhancements in efficiency compared to traditional methods. These advanced techniques minimize both the extraction time and solvent usage, making the process more sustainable and cost-effective (34). These modern methods are crucial for enhancing productivity, improving the quality and quantity of the output, and reducing costs and environmental impact.

Efficiently optimizing the polyphenol extraction process is crucial for enhancing productivity, improving the quality and quantity of the output, and reducing costs and environmental impact. Key aspects to consider during optimization include the specific types of polyphenols, raw material characteristics, extraction costs, the intended application of the final product, and the overall ecological consequences (35). Conducting laboratory-scale optimization followed by validation for scaled production is crucial to determine the most suitable extraction methods for specific applications. By thoroughly evaluating these factors (Table 1C), researchers can significantly improve the effectiveness of polyphenol extraction, ensuring the process is scientifically rigorous and practically viable. This approach allows for the development of optimized extraction protocols that maximize polyphenol yield and quality while being cost-effective and environmentally sustainable.

## 3 Biological activity

### 3.1 Antibacterial activity

**Figure 1:**
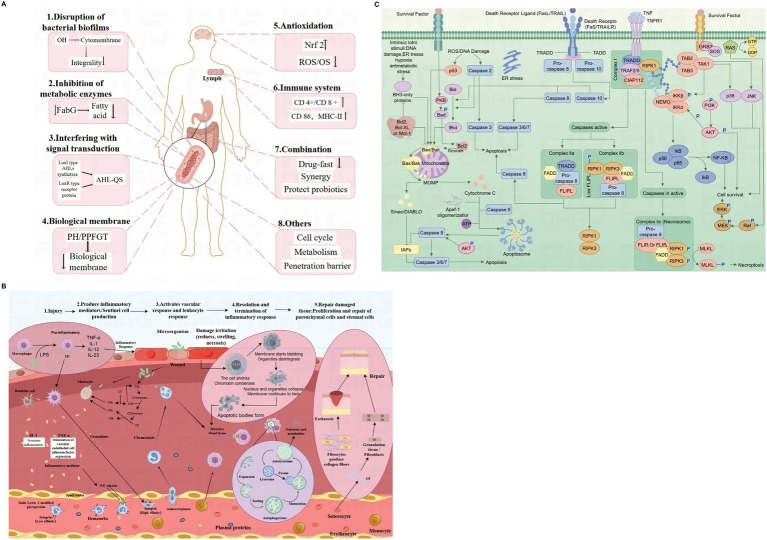
Mechanisms of polyphenols in biological systems: antibacterial mechanisms of polyphenols: disruption of biofilms, enzyme inhibition, signal transduction interference, and immune modulation **(A)**. Cellular and molecular mechanisms of inflammation and tissue repair **(B)**. Mechanisms of mitochondrial oxidative stress and its impact on apoptosis and survival pathways **(C)**.

Polyphenols exhibit various antibacterial mechanisms (Figure 1A), closely tied to their structure, chemical composition, and lipophilicity (36). These compounds can damage bacterial cell membranes, impairing their structure and function (37). For instance, polyphenols can interact with bacterial cell membranes, leading to structural damage and leakage of cellular contents, which is facilitated by the hydroxyl groups in their structure that form hydrogen bonds with the membrane (38). Studies have shown that this interaction is proportional to the number of hydrogen bond donors, and the presence of alkyl groups in the aromatic ring enhances the antibacterial effect by producing phenoxy free radicals (39, 40). Polyphenols also disrupt bacterial intercellular communication systems, known as quorum sensing (QS), which regulate phenotypic traits in a density-dependent manner. Rose tea extract, for instance, significantly inhibits QS and reduces the motility of *Escherichia coli* and *Pseudomonas aeruginosa* (41). Tannic acid, ellagic acid, and epigallocatechin gallate (EGCG) specifically inhibit N-acyl-homoserine lactones (AHLs)-mediated QS in Gram-negative bacteria, blocking bacterial signaling and coordinating activities critical for infection and survival (42).

Furthermore, polyphenols possess significant anti-biofilm properties (43). Biofilms are structural communities of microbial cells encased in extracellular polysaccharides, providing a protective barrier against host immune responses and antibiotics. Polyphenols inhibit biofilm formation without affecting bacterial cell growth, thus reducing the protective effects of the biofilm and decreasing bacterial resistance to antibiotics (37). This has been demonstrated in studies using Padma heptaplex (PH) and polyphenol extracts from green tea (PPFGT), where therapeutic effects were enhanced (44).

Polyphenols also inhibit key enzymes involved in bacterial growth and metabolism. For example, tea polyphenols affect the membrane proteins of *P. aeruginosa* by inhibiting enzymes in the tricarboxylic acid cycle, fatty acid biosynthesis, protein biosynthesis, and DNA metabolism (45). Catechins inhibit the antibacterial activity of DNA gyrase, while apigenin inhibits both DNA gyrase and hydroxyacyl-acyl carrier protein dehydratase activity (46).

Moreover, polyphenols can decrease bacterial outer membrane permeability, leading to the loss of chemiosmotic control and cell death. The effectiveness of polyphenols as antimicrobial agents depends on factors such as chemical structure, concentration, bacterial species, and environmental conditions, with varied efficacy in laboratory versus food settings (47). Despite these variables, polyphenols offer significant advantages in antibacterial food packaging by inhibiting microbial growth with minimal impact on food quality. The diversity of plant-derived polyphenols reduces the likelihood of contributing to antimicrobial resistance and may even reverse antibiotic resistance, thereby reducing human exposure to resistant bacteria (48). This positions polyphenols as promising natural alternatives to synthetic antioxidants in food products, addressing consumer concerns about the carcinogenicity of synthetic compounds and enhancing the safety and quality of food.

### 3.2 Anti-inflammatory effect

Inflammation is the body’s defensive response to injury or infection, involving several stages (Figure 1B). Various injurious agents, such as viruses, bacteria, and microorganisms, damage tissues or cells, prompting nearby sentinel cells (macrophages, mast cells, dendritic cells) to recognize these harmful agents or necrotic materials (49). This recognition triggers the production of inflammatory mediators, initiating vascular responses and leukocyte reactions. Consequently, leukocytes and plasma proteins exude from the blood circulation surrounding the injury site. These white blood cells and plasma proteins then arrive at the site of damage, where they work to dilute, neutralize, and eliminate harmful substances. Phagocytes engulf apoptotic tissues and cells, producing growth factors (GF) and cytokines that stimulate fibroblasts and stromal cell proteins, thereby accelerating the repair of injured sites. Additionally, in response to inflammatory mediators, monocytes in the blood differentiate into macrophages, supplementing the inflammatory response process.

Many diseases are inextricably linked to inflammation, including cancer, type II diabetes, obesity, arthritis, and neurodegenerative diseases (50). Polyphenols can mitigate inflammatory responses through various pathways. For instance, they inhibit the expression of proinflammatory genes (30), reduce the production of inflammatory mediators, and suppress the activity of enzymes involved in inflammatory processes, such as cyclooxygenase, lipoxygenase, MAPK (mitogen-activated protein kinase), and IKK (κ kinase inhibitor) (29, 51, 52). Table 1C summarizes the anti-inflammatory mechanisms of polyphenols.

Polyphenols inhibit the production of inflammatory mediators by inflammatory cells, such as macrophages and monocytes, including prostaglandin E2 (PGE2), tumor necrosis factor α (TNF-α), interleukin (IL-1β, IL-6, etc.), and other cytokines (26). This action on macrophages plays a crucial role in the inflammatory response. Furthermore, polyphenols regulate intracellular signaling pathways by inhibiting the activation of NF-κB (nuclear factor κB), a key transcription factor that regulates the expression of inflammatory genes involved in cellular immunity, inflammation, stress, proliferation, and apoptosis (27). They inhibit NF-κB activation and reduce the expression of inflammatory genes by affecting IKK activation, regulating oxidant levels, or interfering with the binding of NF-κB to DNA (53). Polyphenols also modulate MAPK signaling and arachidonic acid signaling to reduce the inflammatory response at various levels, including blocking the release of TNF-α (53).

Additionally, the antioxidant properties of polyphenols help neutralize free radicals and reactive oxygen species (ROS) produced during inflammation, which cause cellular damage. By eliminating these harmful substances, polyphenols alleviate oxidative stress and the associated inflammatory responses (28). The anti-inflammatory mechanisms of polyphenols render them promising therapeutic agents for treating and the treatment and prevention of inflammatory diseases. However, the anti-inflammatory effect of polyphenols may differ depending on various factors, including their chemical structure, dose, mode of administration, and individual differences (54). Future research should focus on identifying the most effective polyphenols and their optimal dosages.

### 3.3 Antioxidant performance

Additionally, polyphenols play a crucial role in enhancing the host immune system. Macrophages, which are pivotal in the immune response, perform functions such as phagocytosis, antigen presentation, and immune regulation. Resveratrol, for instance, has been shown to enhance macrophage phagocytic activity and antigen-presenting capability, elevate the expression of CD86 and MHC-II, activate the TLR4/NF-κB/JNK signaling pathway, and increase the proportion of CD4+ and CD8+ T cells in peripheral blood (55, 56), thereby amplifying the immune response. Lymphocytes contribute to immunity by producing antibodies via B cells, which neutralize or mark bacteria for destruction. T cells not only bolster the immune response and directly kill infected cells but also differentiate into memory cells, providing long-term immune protection. This establishes a specific and enduring immune defense against bacterial infections.

Chemically synthesized antioxidants such as butylated hydroxyanisole (BHA), butylated hydroxytoluene (BHT), propyl gallate (PG), and tert-butylhydroquinone (TBHQ) are commonly utilized (57). However, the extensive use of these synthetic antioxidants has raised concerns about their potential adverse effects. High doses of chemically synthesized antioxidants have been shown to cause DNA damage, endocrine disruption, and carcinogenic effects (58–60). For example, BHA can interfere with endocrinology, induce cell cycle arrest, and affect male fertility (61). Similarly, BHT has been linked to effects on mitochondrial and endoplasmic reticulum homeostasis, stalling the cell cycle (62). Moreover, both BHA and BHT have been associated with direct carcinogenic effects (63).

Given these concerns, there is a growing need to identify natural antioxidants that can serve as viable alternatives to traditional synthetic antioxidants. The process of fat oxidation, for example, commences when hydrogen atoms of an unsaturated fatty acid (RH) are removed by an active molecules (such as the hydroxyl radical •OH), forming an alkyl radical (R•). This alkyl radical subsequently forms a peroxy radical (ROO•), which removes hydrogen atoms from another fatty acid molecule, resulting in the formation of new alkyl radicals and lipid peroxides (ROOH). The two free radicals eventually interact to form a non-free radical stable molecule, such as a dimer or polymer, thus concluding the oxidation process (57). The addition of antioxidants interferes with this process, retarding oxidation.

The process of protein oxidation is similar to lipid oxidation, but the free radicals formed in the initial stage are protein radicals, which undergo further oxidative reactions to ultimately form non-radical products (57). The final product of protein oxidation can be a carbon–carbon cross-linked derivative (64, 65). The distinction between lipid and protein oxidation is also reflected in the resulting oxidation products. Lipid oxidation can alter taste and odor, whereas protein oxidation can affect juice, tenderness, and color.

Figure 1C shows some of the pathways of apoptosis and survival. During apoptosis, the cell first undergoes a reduction in size, coalescence of genetic material, and eventual breakdown into apoptotic vesicles, which are ultimately removed by phagocytes. Several natural products have demonstrated significant antioxidant potential. Studies show that many vegetables and fruits, such as sorghum (66), rosehips (67), olive oil and olives (68), grape seeds, cherries, berries, pomegranates, parsley, artichokes, and kale (69), as well as whole grains (such as rice) (70), and catechins and theaflavins in tea, possess considerable antioxidant properties. Some studies have explored the potential of these compounds for food preservation, yielding encouraging results. For instance, rosemary, which is rich in caffeic acid, rosmarinic acid, flavonoids, and phenolic diterpenes, has shown the highest antioxidant and antibacterial effects in experiments with cottage cheese (71). Additionally, rosemary extract demonstrated similar protein antioxidant properties to BHT in the preservation of refrigerated pig liver (72). Guava leaf extract, when incorporated into fresh pork sausage, inhibited fat oxidation compared to a control group with BHT added (73). Similarly, adding bee pollen to sausages stored at low temperatures for a month significantly reduced lipid peroxidation (74).

However, certain limitations exist, such as the instability of astaxanthin and carotene upon exposure to light, which diminishes their antioxidant efficacy (75, 76). The antioxidant capacity of polyphenols is attributed to the presence of hydroxyl groups in their structure, with the number and position of these groups being crucial determinants of their antioxidant potential. The antioxidant effect of polyphenols is primarily realized through several mechanisms. Polyphenols engage in free radical scavenging by directly reacting with free radicals, including oxygen radicals (ROS), nitrogen radicals (RNS), and carbon-centered radicals. They neutralize these free radicals through the donation of hydrogen atoms (hydrogen atom transfer, HAT) or electrons (electron transfer, ET), thereby preventing the chain reactions initiated by these radicals (77). Polyphenols possess multiple hydroxyl groups that enable them to form stable chelates with transition metal ions, thereby reducing the generation of free radicals catalyzed by these metals. For instance, curcumin is capable of chelating metal ions such as Cu^2+^ and Fe^2+^ (53), while quercetin chelates ferrous ions (78).

Polyphenols are also capable of inhibiting the activity of specific oxidases, such as lipoxygenase (LOX) and cyclooxygenase (COX) (79). These enzymes are responsible for catalyzing the production of active oxides during inflammation and oxidative stress, which in turn generate inflammatory mediators like leukotrienes and nitric oxide (79). Polyphenols exert their effects by enhancing the antioxidant enzyme system, wherein they activate or upregulate the activity of intracellular antioxidant enzymes such as superoxide dismutase (SOD), glutathione peroxidase (GPx), and catalase (CAT). These enzymes are pivotal in the detoxification of reactive oxygen species (ROS). For example, curcumin has been demonstrated to augment the activity of SOD, GPx, and catalase, consequently mitigating peroxide toxicity (80).

These antioxidant mechanisms act in concert to render polyphenols compelling candidates for the prevention and treatment of oxidative stress-related diseases. Nevertheless, the antioxidant effect of polyphenols is susceptible to modulation by a number of factors, including their chemical structure, concentration, presence form, bioavailability, and other variables. Polyphenols can be employed as antioxidants due to their antioxidant properties. For instance, resveratrol has been demonstrated to play a role in protecting cellular components from oxidative damage and reducing the risk of various degenerative diseases (81).

The figure effectively illustrates the potential of polyphenols in therapeutic applications, including antibacterial treatments, anti-inflammatory interventions, and protection against oxidative stress-related diseases. Future research should focus on identifying specific polyphenol compounds that are most effective in these roles and exploring their bioavailability and efficacy in clinical settings. Understanding the synergies between different polyphenols and their combined effects on these pathways could also open new avenues for developing polyphenol-based therapeutics.

## 4 Conclusion

Optimizing the extraction processes of polyphenols to enhance efficiency, yield, and quality while reducing environmental impact is aligned with the principles of sustainable and green processing. The challenges of incorporating polyphenols into food products must be addressed with a focus on food safety, sensory quality, and compliance with health standards. Sustainable practices in food processing, including the use of renewable resources, energy-efficient technologies, and waste reduction techniques, are essential for maximizing the benefits of polyphenols. Additionally, advanced analytical techniques such as spectroscopy, chromatography, and molecular diagnostics are crucial for accurately evaluating polyphenol content and efficacy.

This review emphasizes the imperative for sustained research into the intricate mechanisms of polyphenols. Innovation in the application of polyphenols can be driven by fostering cooperation and knowledge sharing among academics, scientists, and industrial experts. This collaborative effort is aimed at promoting the sustainability of advanced food processing methods, ensuring continuous improvement in both processing techniques and product quality, and embracing the principles of the green revolution. Overall, plant polyphenols have the potential to transform the food industry by enhancing the safety, traceability, and authenticity of food products, while facilitating sustainable processing methods and improving food quality assessment.
